# Sea Spike Suppression Method Based on Optimum Polarization Ratio in Airborne SAR Images

**DOI:** 10.3390/s21093269

**Published:** 2021-05-09

**Authors:** Yawei Zhao, Jinsong Chong, Yan Li, Kai Sun, Xue Yang

**Affiliations:** 1 National Key Lab of Microwave Imaging Technology, Beijing 100190, China; zhaoyawei17@mails.ucas.ac.cn (Y.Z.); liyan1603@mails.ucas.ac.cn (Y.L.); sunkai181@mails.ucas.ac.cn (K.S.); yangxue19@mails.ucas.ac.cn (X.Y.); 2 Aerospace Information Research Institute, Chinese Academy of Sciences, Beijing 100190, China; 3 School of Electronics, Electrical and Communication Engineering, University of Chinese Academy of Sciences, Beijing 100049, China

**Keywords:** SAR, sea spike suppression, optimum polarization ratio

## Abstract

In the condition of ocean observation for high-resolution airborne synthetic aperture radar (SAR), sea spikes will cause serious interference to SAR image interpretation and marine target detection. In order to improve the ability of target detection, it is necessary to suppress sea spikes in SAR images. However, there is no report on sea spike suppression methods in SAR images. As a step forward, a sea spike suppression method based on optimum polarization ratio in airborne SAR images is proposed in this paper. This method is only applicable to the situation where VV and HH dual-polarized SAR data containing sea spikes are acquired at the same time. By calculating the optimum polarization ratio, this method further obtains the difference image of the panoramic area accomplishing sea spike suppression. This method is applied to a field airborne X-band SAR data, including ocean waves, oil spills and ships. The results show that the sea spikes are well suppressed, the contrast of ocean waves and the contrast of oil spills are improved, and the false alarm rate of ship detection is reduced. The discussions on these results demonstrate that the proposed method can effectively suppress sea spikes and improve the interpretability of SAR images.

## 1. Introduction

Synthetic aperture radar (SAR) is widely applied to the field of ocean remote sensing with obvious advantages of all-day, all-weather, high resolution and wide swath coverage. With the rapid development of airborne SAR technology, the resolution of airborne SAR images becomes higher every day. High-resolution airborne SAR brings us more plentiful target details, but sea spikes are prone to appear when observing the ocean. It seriously affects SAR ocean image interpretation and marine target detection [1,2,3], which brings new challenges to the marine application of SAR images.

Sea spikes manifest as sporadic movement or stationary "targets" randomly distributed at different distances and angles [4]. From the experiments and numerical simulations, breaking waves have been suggested as the main contributor to sea spikes [5,6,7]. Generally speaking, as the wind speed increases, the ocean wave height and rms slope will increase [8]. When the inclination of the sea surface increases to the certain angle, the surge loses its original balance, and the peak of the wave sprays sharply. This phenomenon is called the wave breaking and is the main reason of radar spikes forming [8]. The existence of sea spikes will cause false alarms of target detection [9,10], and sea spike suppression has always been a hot issue in the radar world.

Most of the sea spike suppression methods focus on intelligent pixel processing X-band (IPIX) datasets, which can be divided into three major categories. A typical method of the first type is the time-domain suppression method [11,12]. The core idea of this method is to identify the characteristics of sea spikes in time-domain, thereby suppressing sea spikes. The second is based on the frequency domain filtering method, which is typically represented by a block-adaptive clutter suppression filter [13]. The third is the decomposition method of the feature matrix [14].

At present, there is no report on sea spike suppression methods in SAR images. Moreover, because SAR data acquisition and signal processing methods are different from IPIX radar, the sea spike suppression methods cannot be directly applied to SAR data.

In order to solve this problem, a sea spike suppression method based on optimum polarization ratio in airborne SAR images is proposed in this paper, which is designated for the single-look complex (SLC) data of dual-polarized (VV and HH polarization) SAR. Firstly, this method selects a background sub-block from the dual-polarized data. Then, a sequence of polarization ratios is generated by traversing the theoretical interval of polarization ratio. Subsequently, utilising the average intensity of the dual-polarized background sub-block and the sequence of polarization ratios, a sequence of difference images of the background sub-block is calculated. To acquire the optimum polarization ratio, the sequence of difference images is compared. Finally, the method utilizes the optimum polarization ratio to calculate the difference image of the panoramic area, and then the image of sea spike suppression can be obtained. The airborne X-band SAR data is employed to obtain the image of sea spike suppression with the proposed method. The three aspects, namely ocean wave contrast, oil spill contrast and ship detection results, are analyzed, which verify the effectiveness of this method.

The rest of the paper is organized as follows. In Section 2, the proposed method is introduced in detail. In Section 3, the effectiveness of the proposed method is verified by field data. Besides, the results of the method are analyzed through ocean wave contrast, oil spill contrast and ship detection results. In Section 4, the proposed method and the spectrum filtering method are compared, and the applicability of the proposed method is analyzed in this section. Finally, conclusions are made in Section 5.

## 2. Sea Spike Suppression Method Based on Optimum Polarization Ratio in Airborne SAR Images

**Figure 1:**
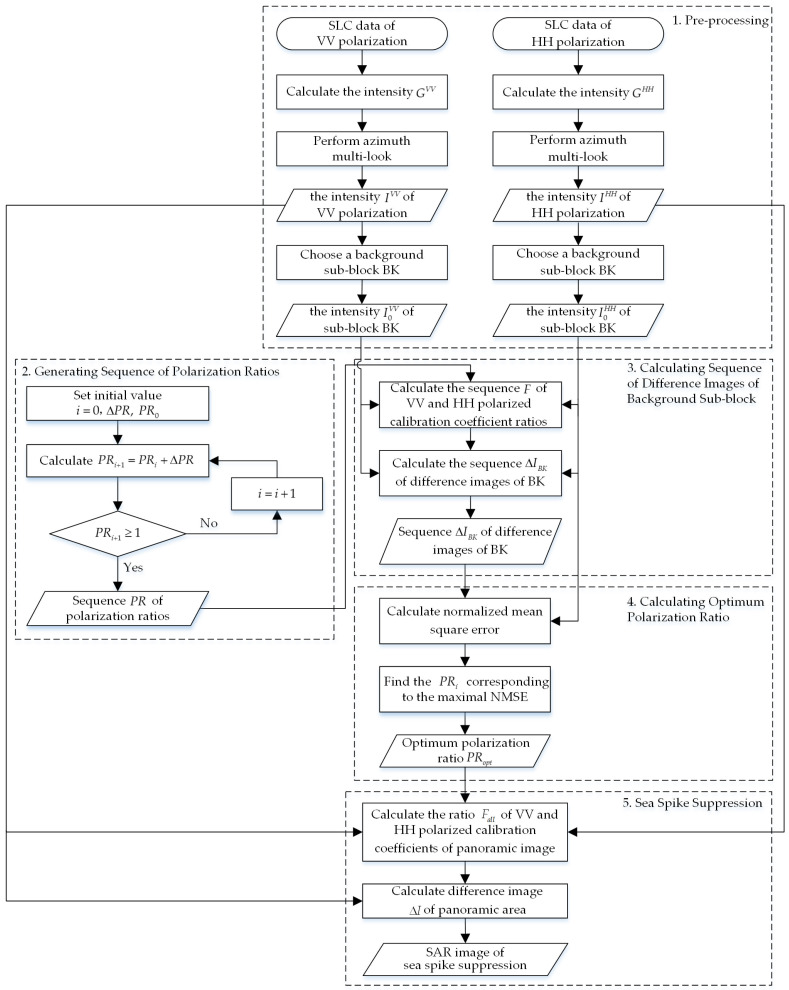
Flow chart of the proposed method.

In this section, a sea spike suppression method based on optimum polarization ratio in airborne SAR images is proposed, which is designated for the single-look complex (SLC) data of dual-polarized airborne SAR. The flow chart of the proposed method is shown in Figure 1. As can be seen, the method can be divided into five parts: pre-processing, generating sequence of polarization ratios, calculating sequence of difference images of background sub-block, calculating optimum polarization ratio and sea spike suppression. The five parts are introduced below.

### 2.1. Pre-Processing

The intensities of the VV and HH polarized SLC data are calculated and denoted as $G^{VV}$ and $G^{HH}$, respectively. These SLC images are dealt through the azimuth multi-look processing, thus the influence of speckle noises can be reduced. The number $L_{azi}$ of looks is determined by the ratio of the range resolution and the azimuth resolution, which can be expressed as

(1) $$L_{azi}=\left\lfloor \frac{R_{bin}}{X_{bin}}\right\rfloor$$

where $\left\lfloor \cdot \right\rfloor$ denotes the function of round-down, $R_{bin}$ indicates the size of the range resolution cell of the SLC images, and $X_{bin}$ indicates the size of the azimuth resolution cell. After the azimuth multi-look processing, the intensities of VV and HH polarization are $I^{VV}$ and $I^{HH}$, respectively.

From $I^{VV}$ and $I^{HH}$, we select a M∗N background sub-block, i.e., BK, according to the following two constraints: (1) sea spikes must exist in BK; and (2) no artificial targets exist in BK. SAR ocean images generally contain man-made targets such as ships and oil spills, which will influence the subsequent processing. For this reason, a uniform background sub-block containing only sea spikes is selected for subsequent calculating the sequence of difference images of background sub-block and optimum polarization ratio.

### 2.2. Generating Sequence of Polarization Ratios

The polarization ratio of the background sub-block BK is defined as the ratio of HH polarized backscatter coefficient $\sigma_{BK}^{HH}$ to VV polarized backscatter coefficient $\sigma_{BK}^{VV}$, i.e.,

(2) $$PR_{BK}=\frac{\sigma_{BK}^{HH}}{\sigma_{BK}^{VV}}$$

The fundamental relationship between the backscattering coefficient of background sub-block and SAR image intensity is defined as the equation [15]

(3) $$\sigma_{BK}^{pp}=\frac{I_{BK}^{pp}}{K_{pp}}$$

where pp is the polarization mode, and $K_{pp}$ is the calibration coefficient for the pp polarized background sub-block BK. According to the literature [16], the calibration coefficient is a function of slant range. Since the slant range within the background sub-block BK slowly changes, the calibration coefficient here can be approximated as a constant $K_{pp}$.

Thus, substituting Equation (3) into Equation (2), it can be get that

(4) $$PR_{BK}=\frac{K_{VV}I_{BK}^{HH}}{K_{HH}I_{BK}^{VV}}$$

$F_{BK}$ is defined as the ratio of the calibration coefficient of VV polarization to that of the HH polarization in the background sub-block BK

(5) $$F_{BK}=\frac{K_{VV}}{K_{HH}}$$

Hence, Equation (4) can be simplified to

(6) $$PR_{BK}=\frac{F_{BK}I_{BK}^{HH}}{I_{BK}^{VV}}$$

Breaking waves have been suggested as the main contributor to sea spikes [5,6,7]. In the case of high frequency (X, Ku, Ka band), SAR ocean surface imaging needs to consider the impact of breaking waves on the polarization ratio. Therefore, this paper chooses the theoretical model of the polarization ratio to analyze, which considers the impact of breaking waves [17]. The ratio of VV polarized backscatter coefficient to HH polarized backscatter coefficient is expressed as

(7) PRVVVVHHHH=PRBragg1−αsin2θ1+αsin2θ

where

(8) $$PR_{Bragg}=\frac{{\left(1+\sin^{2}\theta \right)}^{2}}{\cos^{4}\theta }$$

where $PR_{Bragg}$ is the ratio of VV polarized backscatter coefficient to HH polarized backscatter coefficient for the two-scale Bragg scattering. The Bragg wavelength depends on the local incidence angle: λBragg=λλ2sinθ2sinθ, where λ is the radar wavelength [18]. θ is the incidence angle and α is the tuning parameter.

Therefore, the ratio of HH polarized backscatter coefficient to VV polarized backscatter coefficient can be expressed as

(9) $$PR_{model}=\frac{\cos^{4}\theta \left(1+\alpha \sin^{2}\theta \right)}{{\left(1+\sin^{2}\theta \right)}^{2}\left(1-\alpha \sin^{2}\theta \right)}$$

**Figure 2:**
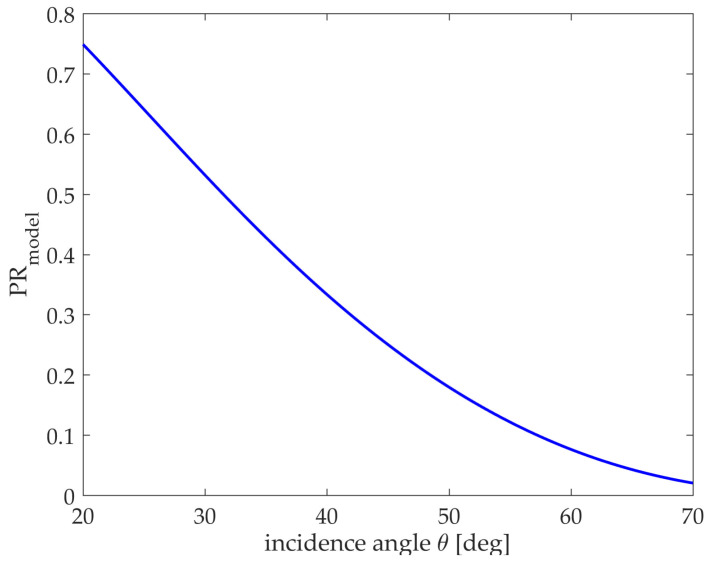
Polarization ratio curve with θ(α=0.77).

In the literature [17], the X-band SAR data is fitted to obtain α=0.77, which is under the conditions of medium wind speed and cross wind direction. At present, the curve of polarization ratio $PR_{model}$ variation in the model with the incidence angle θ is shown in Figure 2.

Airborne SAR generally works at medium incidence angle. According to Equation (9), when the medium incidence angle changes a little, the polarization ratio is almost unchanged with different values of α. For example, when α=0.77, if the incidence angle is shifted $\pm 1^{\circ }$ around $\theta =40^{\circ }$, the value of the polarization ratio is approximately shifted ∓0.018. Therefore, it can be assumed that the polarization ratio in the background sub-block BK is a constant. Combined with Equation (6), the polarization ratio ${\bar{PR}}_{BK}$ of the background sub-block BK is expressed as

(10) $${\bar{PR}}_{BK}=\frac{F_{BK}\langle I_{BK}^{HH}\rangle }{\langle I_{BK}^{VV}\rangle }$$

where $\langle \cdot \rangle$ represents the averaging function. $\langle I_{BK}^{pp}\rangle$ can be written as

(11) $$\langle I_{BK}^{pp}\rangle =\frac{\sum_{m=1}^{M}\sum_{n=1}^{N}I_{BK}^{pp}\left(m,n\right)}{MN}$$

where $\left(m,n\right)$ is the coordinates of a pixel, satisfying 1⩽m⩽M and 1⩽n⩽N.

In practice, atmospheric turbulences can produce large motion errors in airborne SAR systems, which introduce radiometric distortions in the SAR data [19]. Besides, it is quite difficult to derive absolutely calibrated backscattering coefficient of ocean due to the system noise [20]. Therefore, airborne SAR data is usually uncalibrated when observing the ocean. In this paper, we intend to generate a sequence of polarization ratios within the theoretical range to approach as close as possible to the polarization ratio ${\bar{PR}}_{BK}$ of the background sub-block in subsequent processing.

The redistribution of radar returns between short-scale Bragg waves and wave breaking is characterized by the value of ${\bar{PR}}_{BK}$ in the background sub-block BK [21]. This ratio is minimal for the pure Bragg scattering and approaches 1 when breaking wave component dominates [21]. Therefore, the polarization ratio ${\bar{PR}}_{BK}$ is in the range of $\left(0,1\right)$.

By traversing the theoretical interval $\left(0,1\right)$, the sequence of polarization ratios will be generated. For initialization, the iterating times *i* is set as 0. For higher precision, the step-size ΔPR is defined between 0 and 0.1, and the initial value $PR_{0}$ of the polarization ratio is constrained in the range of 0.01–0.05. Thus, the traverse is conducted through $PR_{i+1}=PR_{i}+\Delta PR$(where $i=0,1,\cdots ,\left\lfloor \frac{1-PR_{0}}{\Delta PR}\right\rfloor$) and the sequence PR of polarization ratios is obtained as

(12) $$PR=\left[PR_{i}\right],i=0,1,\cdots ,\left\lfloor \frac{1-PR_{0}}{\Delta PR}\right\rfloor$$

### 2.3. Calculating Sequence of Difference Images of Background Sub-Block

Polarization difference (PD) can remove the wave breaking contribution [21,22], so it will remove sea spikes effectively. The polarization difference of background sub-block BK can be described as

(13) $$PD_{BK}=\sigma_{BK}^{VV}-\sigma_{BK}^{HH}$$

Substituting Equations (3) and (5) into Equation (13), we can obtain

(14) $$PD_{BK}=\frac{I_{BK}^{VV}}{K_{VV}}-\frac{I_{BK}^{HH}}{K_{HH}}=\frac{1}{K_{VV}}\left(I_{BK}^{VV}-F_{BK}I_{BK}^{HH}\right)$$

Equation (14) indicates that $PD_{BK}$ is proportional to $\left(I_{BK}^{VV}-F_{BK}I_{BK}^{HH}\right)$, so the value of $\left(I_{BK}^{VV}-F_{BK}I_{BK}^{HH}\right)$ can be used to suppress sea spikes. Equation (10) implies that $F_{BK}$ can be calculated by the polarization ratio ${\bar{PR}}_{BK}$ of the background sub-block BK

(15) $$F_{BK}={\bar{PR}}_{BK}\frac{\langle I_{BK}^{VV}\rangle }{\langle I_{BK}^{HH}\rangle }$$

Thus, combined with the ratio IBKVVIBKVVIBKHHIBKHH, the sequence *F* of VV and HH polarized calibration coefficient ratios corresponding to the sequence PR of polarization ratios is calculated by

(16) $$F=PR\frac{\langle I_{BK}^{VV}\rangle }{\langle I_{BK}^{HH}\rangle }$$

where the sequence *F* is described as

(17) $$F=\left[F_{i}\right],i=0,1,\cdots ,\left\lfloor \frac{1-PR_{0}}{\Delta PR}\right\rfloor$$

Then, the sequence $\Delta I_{BK}$ of difference images between $I_{BK}^{VV}$ and $F\cdot I_{BK}^{HH}$ is calculated by

(18) $$\Delta I_{BK}=I_{BK}^{VV}-F\cdot I_{BK}^{HH}$$

where the sequence $\Delta I_{BK}$ is expressed as

(19) $$\Delta I_{BK}=\left[\Delta I_{BK_{i}}\right],i=0,1,\cdots ,\left\lfloor \frac{1-PR_{0}}{\Delta PR}\right\rfloor$$

### 2.4. Calculating Optimum Polarization Ratio

In this paper, we define the polarization ratio that is the closest to the ${\bar{PR}}_{BK}$ as the optimum polarization ratio $PR_{opt}$. When $PR_{i}=PR_{opt}$, $F_{i}\to F_{BK}$ and the difference image $\Delta I_{BK_{i}}$ of background sub-block BK correspondingly can be acquired by Equation (18).

(20) $$\Delta I_{BK_{i}}\approx I_{BK}^{VV}-F_{BK}I_{BK}^{HH}$$

Combining Equations (14) and (20), it can be found that $\Delta I_{BK_{i}}$ is proportional to $PD_{BK}$ at this time. This means when $PR_{i}=PR_{opt}$, the difference image is regarded as the result of sea spike suppression. Thus, the optimum polarization ratio $PR_{opt}$ will be obtained by comparing the sequence $\Delta I_{BK}$ of difference images of the background sub-block BK.

Since the effect of sea spikes on HH polarization is greater than those on the VV polarization [23], the normalized mean square error (NMSE) between the HH polarization intensity $I_{BK}^{HH}$ and the sequence $\Delta I_{BK}$ of difference images will be used as the evaluation index to obtain the optimum polarization ratio $PR_{opt}$. The $NMSE_{i}$ corresponding to each difference image $\Delta I_{BK_{i}}$ is calculated as

(21) $$NMSE_{i}=\frac{\sum_{m=1}^{M}\sum_{n=1}^{N}{\left[I_{BK}^{HH}\left(m,n\right)-\Delta I_{BK_{i}}\left(m,n\right)\right]}^{2}}{\sum_{m=1}^{M}\sum_{n=1}^{N}{\left[I_{BK}^{HH}\left(m,n\right)\right]}^{2}}$$

Consequently, these NMSEs are compared one by one. The maximal NMSE reflects the dissimilarity between the corresponding difference image $\Delta I_{BK_{i}}$ and the HH polarized intensity $I_{BK}^{HH}$ reaches largest, and the effect of sea spike suppression is optimum. Thus the optimum polarization ratio $PR_{opt}$ is found.

(22) $$PR_{opt}=\underset{PR_{i}}{\arg\max}NMSE_{i}$$

### 2.5. Sea Spike Suppression

Sea spike suppression will be performed on the panoramic image. By calculating the ratio IVVIVVIHHIHH of the VV and HH polarized data, and substituting it into $F_{all}\approx PR_{opt}\frac{\langle I^{VV}\rangle }{\langle I^{HH}\rangle }$, the difference image $\Delta I=I^{VV}-F_{all}\cdot I^{HH}$ can be calculated. The panoramic image of sea spike suppression can be obtained.

## 3. Validation of the Proposed Method with Field Data

**Figure 3:**
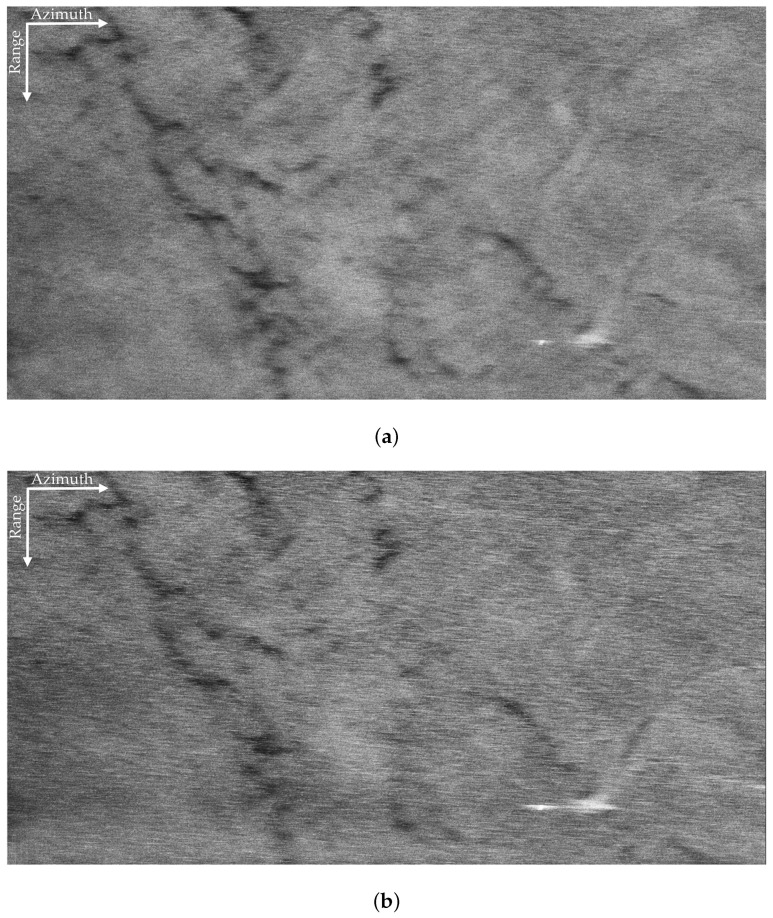
The VV and HH polarized SAR images of X-band. (**a**) The VV polarized SAR image. (**b**) The HH polarized SAR image.

In this section, the proposed method is applied to the field data that were obtained by the Aerospace Information Research Institute, Chinese Academy of Sciences in 2019 covering South China Sea. The parameters of the SAR system are listed in Table 1. The swath width was 1.02 km × 2.16 km (range × azimuth); the incidence angle fell between $\left[46.62^{\circ },\;55.28^{\circ }\right]$; and the size of the SLC resolution cell was 0.17 m × 0.04 m (range × azimuth). The VV and HH polarized SAR images are shown in Figure 3.

**Figure 4:**
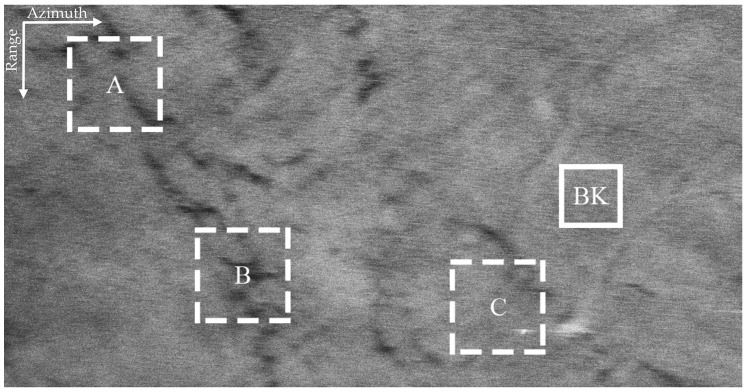
Locations of the four selected sub-blocks.

The VV and HH polarized SAR images in Figure 3 are seriously interfered by sea spikes. For better illustration, we selected four sub-blocks, namely BK, A, B, C. Among them, BK is the background sub-block, A is the ocean waves sub-block, B is the oil spills sub-block, and C is the ship targets sub-block. The sub-block BK is about 170 m × 170 m and does not contain other information apart from sea spikes. Rectangles in Figure 4 identify the locations of the four sub-blocks.

**Figure 5:**
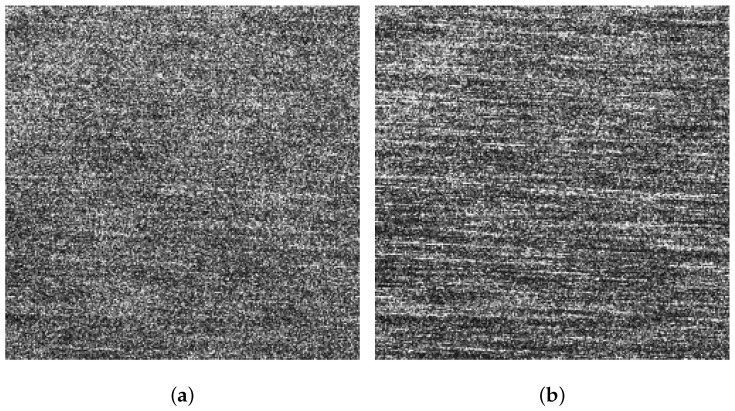
The VV and HH polarized SAR images corresponding to the background sub-block BK. (**a**) The VV polarized SAR image. (**b**) The HH polarized SAR image.

**Figure 6:**
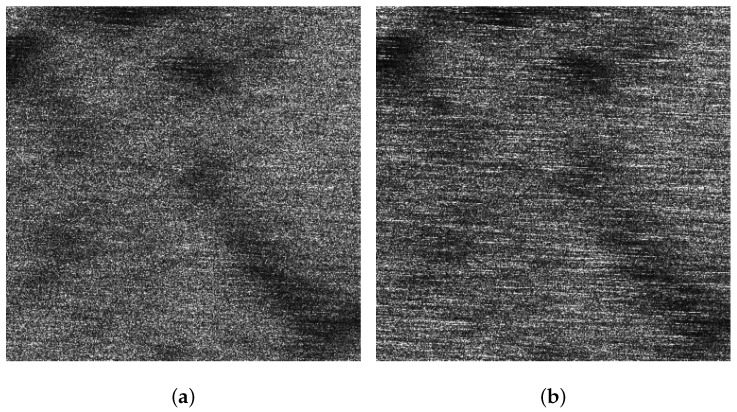
The VV and HH polarized SAR images of sub-block A. (**a**) The VV polarized SAR image. (**b**) The HH polarized SAR image.

**Figure 7:**
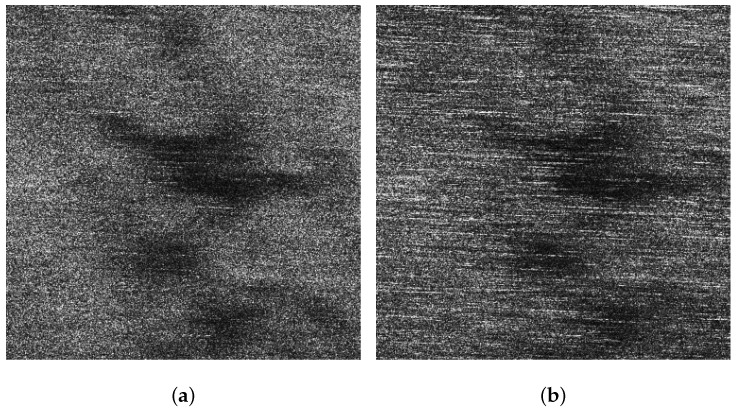
The VV and HH polarized SAR images of sub-block B. (**a**) The VV polarized SAR image. (**b**) The HH polarized SAR image.

**Figure 8:**
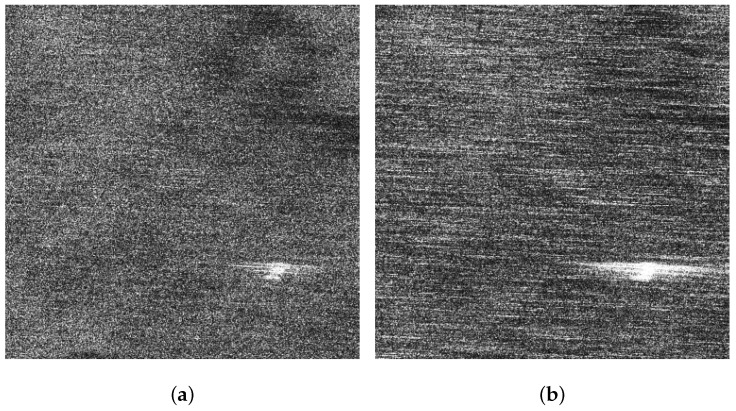
The VV and HH polarized SAR images of sub-block C. (**a**) The VV polarized SAR image. (**b**) The HH polarized SAR image.

Figure 5a,b display the VV and HH polarized images of the background sub-block BK, respectively. It can be found that dual-polarized SAR images are severely affected by the sea spikes. Figure 6a,b are the VV and HH polarized images of the ocean waves sub-block A, respectively. Affected by the sea spikes, both the VV and HH polarized SAR images are hard to distinguish the ocean wave texture. Figure 7a,b are the VV and HH polarized images of the oil spills sub-block B, respectively. Due to the severe impact of the sea spikes, the edge features of the oil spills can barely be delineated. Figure 8a,b are the VV and HH polarized images of the ship targets sub-block C, respectively. The existence of sea spikes in the images can easily cause false alarms during ship detection.

### 3.1. Results of Field Data Processing

**Figure 9:**
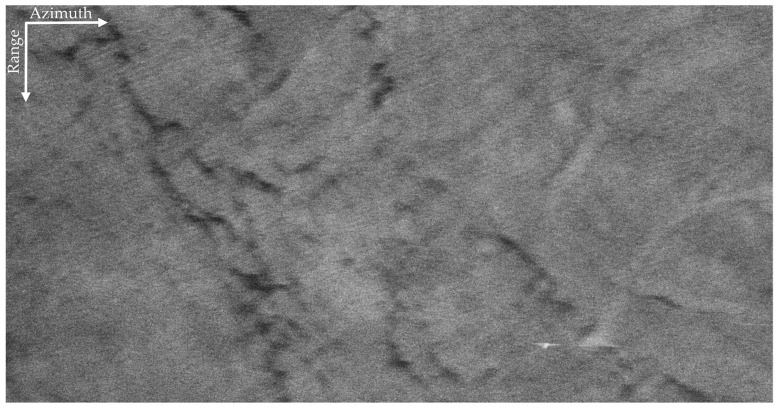
The panoramic image of sea spike suppression.

In pre-processing, the number $L_{azi}$ of azimuth multi-look is 4. For generating the sequence of polarization ratios, we set the step-size ΔPR=0.02, and the initial value of the polarization ratio $PR_{0}=0.02$, with the consideration of both calculation speed and precision. We obtained the final $PR_{opt}$ as 0.36, and applied it to the panoramic image. Figure 9 illustrates the result of sea spike suppression of the panoramic image.

**Figure 10:**
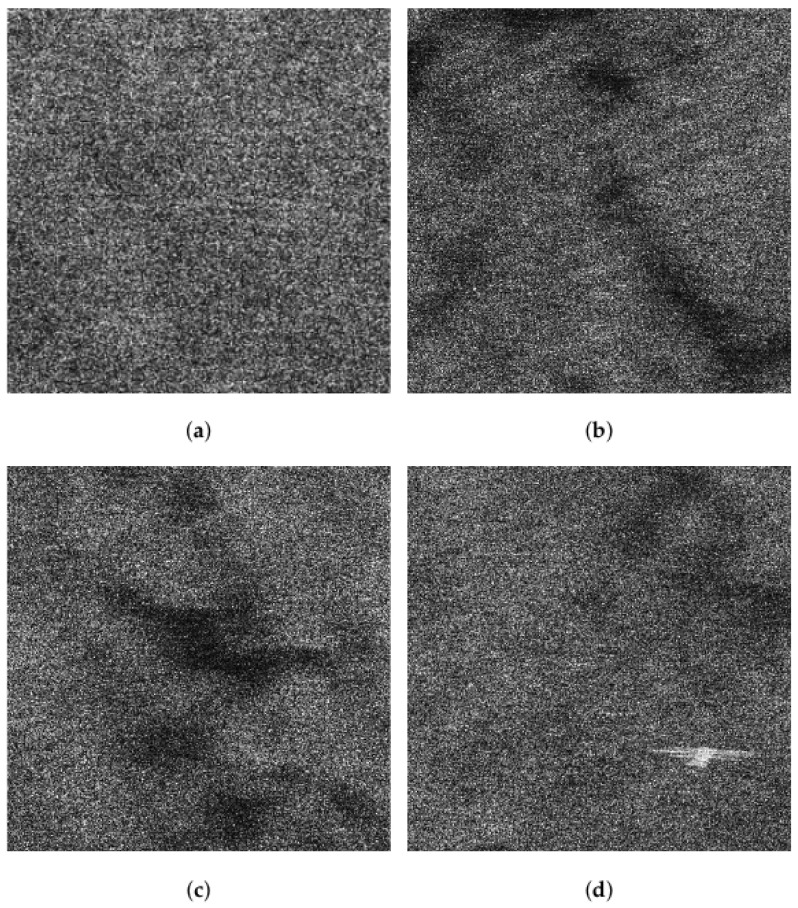
Results of sea spike suppression on sub-blocks BK, A, B, and C. (**a**) Result of sea spike suppression on background sub-block BK. (**b**) Result of sea spike suppression on sub-block A. (**c**) Result of sea spike suppression on sub-block B. (**d**) Result of sea spike suppression on sub-block C.

Comparing Figure 9 with Figure 3a,b, we can infer that the sea spikes have been suppressed, and the quality of the SAR image is improved. In Figure 9, the texture of the ocean waves previously submerged in the sea spikes is highlighted, and the edge features of the oil spills stand out. Figure 10a–d present the detailed results of the sea spike suppression of sub-blocks BK, A, B, and C.

### 3.2. Analysis of the Sea Spike Suppression Results

To further illustrate the effectiveness of this method, this section will analyze the contrast of ocean waves, the contrast of oil spills, and the results of ship detection by sub-blocks A, B, and C, respectively.

#### 3.2.1. Analysis of the Ocean Wave Contrast

(1) Analysis of Image Spectrums

**Figure 11:**
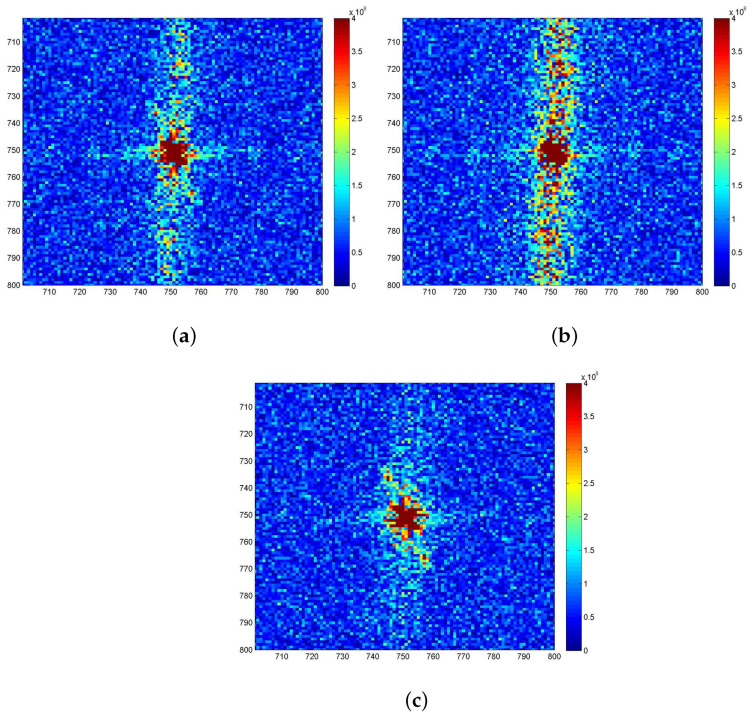
Comparison of image spectrum results of sub-block A. (**a**) The VV polarized image spectrum. (**b**) The HH polarized image spectrum. (**c**) Image spectrum after sea spike suppression.

In this subsection, the ocean wave contrast of sub-block A will be analyzed. Figure 11a–c display the spectrum images of the VV and HH polarized images of sub-block A in Figure 6a,b, as well as the sea spike suppression result of sub-block A in Figure 10b.

By comparison, it is found that due to the influence of sea spikes, the spectrums of the VV polarized ocean waves and the sea spikes are aliased in Figure 11a, which is hard to distinguish. In Figure 11b, the spectrum of the HH polarized ocean waves is completely submerged in that of the sea spike spectrum and the two can barely be separated. After the sea spike suppression, the spectrum of ocean waves can be clearly distinguished in Figure 11c.

(2) Quantitative Analysis of Ocean Wave Contrast

The following is a quantitative analysis of ocean wave contrast of sub-block A. It generally takes the peak-to-background ratio (PBR) as a measurement to analyze the contrast of SAR ocean wave images [24]. PBR is defined as [25]

(23) $$PBR=\frac{{\left(S_{I}\right)}_{\max}}{\langle S_{n}\rangle }$$

where ${\left(S_{I}\right)}_{\max}$ and $\langle S_{n}\rangle$ are the peak value and noise floor of the SAR image spectrum, respectively. The comparison of PBR before and after sea spike suppression on sub-block A is shown in Table 2.

As can be seen from Table 2, the dominant wave contrast of the VV polarized SAR image is higher than that of the HH polarized image. After processing by our method, the PBR achieves 1.49 times higher than that of the HH polarized image and 43.76% higher than that of the VV polarized image, which well demonstrates the effectiveness of this method.

#### 3.2.2. Analysis of the Oil Spill Contrast

(1) Analysis of the Oil Spill Profiles

**Figure 12:**
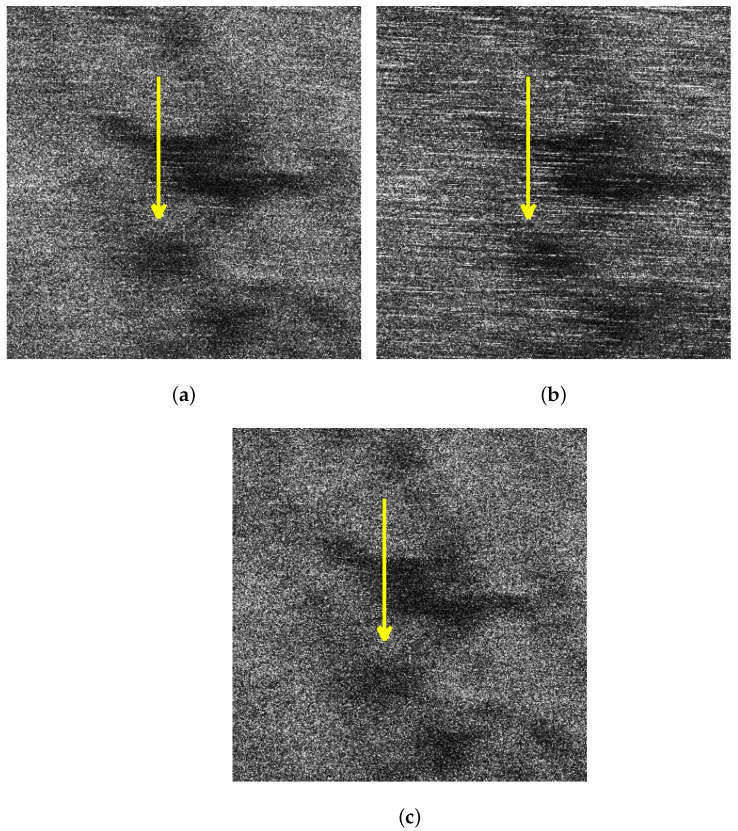
Schematic diagrams of the oil spill profiles in sub-block B. (**a**) Schematic diagram of the VV polarized oil spill profile. (**b**) Schematic diagram of the VV polarized oil spill profile. (**c**) Schematic diagram of the oil spill profile after sea spike suppression.

For better comparison, we delineated the profiles of the oil spills in the VV and HH polarized images of sub-block B as well as the one after sea spike suppression. Figure 12a–c depict the profiles on the images originated from Figure 7a,b and Figure 10c, respectively. Calculating the contrast of profiles shown along the yellow arrows in Figure 12a–c, the equation is expressed as

**Figure 13:**
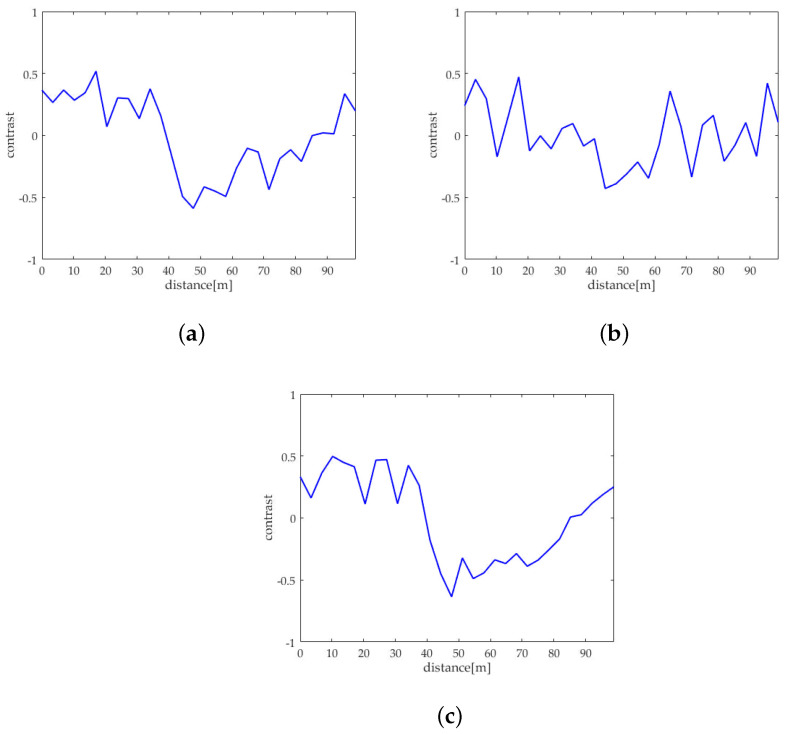
Comparison of contrasts of the oil spill profiles. (**a**) The contrast of the VV polarized oil spill profile. (**b**) The contrast of the HH polarized oil spill profile. (**c**) The contrast of the oil spill profile after sea spike suppression.

(24) CRy=Iy−Iy¯Iy−Iy¯Iy¯Iy¯

where $I_{y}$ represents the intensity of the *y*th pixel on the profile (where 1⩽y⩽Y, *Y* is the total number of the pixels in the profile), and $\bar{I_{y}}$ is the average intensity of the profile. The obtained contrasts corresponding to the profiles in Figure 12a–c are shown in Figure 13a–c, respectively.

The oil spill boundary spread between the distance of 30 m to 50 m. It can be seen that the edge features of the oil spills are extremely inconspicuous under the HH polarization shown in Figure 13b. The edge features of the oil spills are obvious in the profile contrast of VV polarization shown in Figure 13a and the sea spike suppression shown in Figure 13c. In comparison, the edge contrast is more clear under the sea spike suppression. In addition, the dual-polarized SAR images around the distance of 65 m is seriously affected by the sea spikes. After processing by the proposed method in this paper, the sea spikes are well suppressed and the influence of the sea spikes on the oil spill area is reduced.

(2) Quantitative Analysis of Oil Spill Contrast

The following is a quantitative analysis of the oil spill contrast of sub-block B. The contrast between slick-free and slick-covered surfaces in SAR imagery, i.e., ξ, can be defined as the ratio of the mean value of a slick-free background sample $\langle I_{sea}\rangle$ to the mean value of a sample extracted from the slick-covered region $\langle I_{slick}\rangle$, i.e., [26]

(25) $$\xi =\frac{\langle I_{sea}\rangle }{\langle I_{slick}\rangle }$$

The larger the value of ξ, the higher the contrast between slick-free and slick-covered surfaces. Calculate the value of ξ in the profiles shown in Figure 12. The comparison of ξ before and after sea spike suppression on sub-block B is shown in Table 3.

As can be seen from Table 3, the oil spill contrast of the VV polarized SAR image is higher than that of the HH polarized image. After processing by our method, the ξ achieves 34.85% higher than that of the HH polarized image and 6.04% higher than that of the VV polarized image, which shown that the contrast between slick-free and slick-covered is significantly enhanced after suppressing the sea spikes.

#### 3.2.3. Analysis of Ship Detection Results

(1) Analysis of Ship Detection Results 

**Figure 14:**
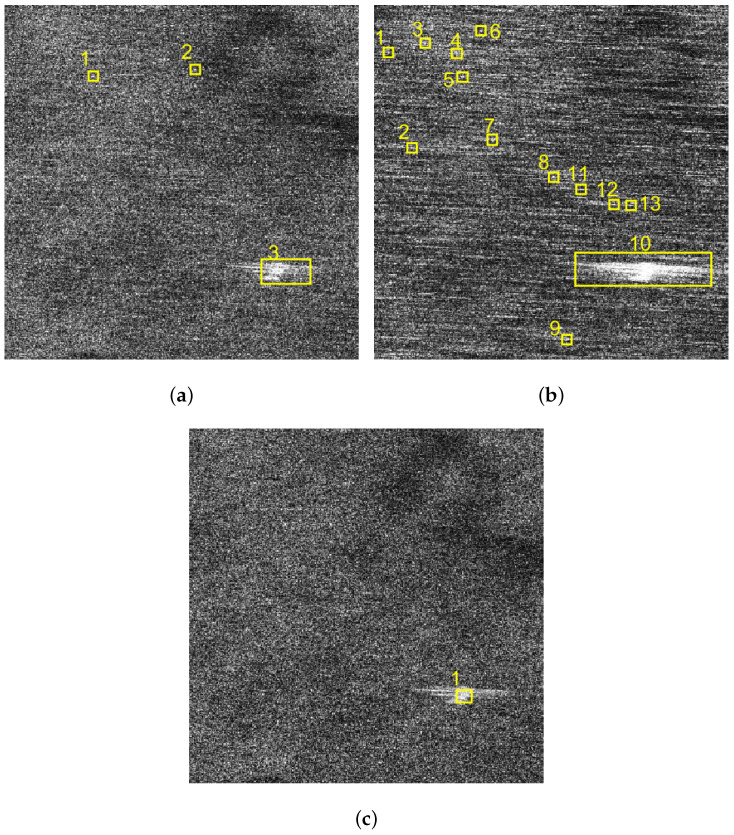
Comparison of ship detection results of images of sub-block C. (**a**) Ship detection result of the VV polarized image. (**b**) Ship detection result of the HH polarized image. (**c**) Ship detection result of the image after sea spike suppression.

In this subsection, a comparison of the ship detection results of sub-block C will be analyzed. In this paper, we adopted the traditional two-parameter CFAR detection method [27] to detect ships in the images of sub-block C. The corresponding detection results of Figure 8a,b and Figure 10d are shown in Figure 14a–c.

Through the detection results, it can be found that the VV polarized SAR image shown in Figure 14a is affected by the sea spikes, and false alarms occur. The HH polarized SAR image shown in Figure 14b is severely affected by the sea spikes, and the false alarm rate of the ship detection is higher than the VV polarized detection. After using the method in this paper, there is no false alarm, as shown in Figure 14c.

(2) Quantitative Analysis of Ship Detection Results 

The figure-of-merit (FoM) is used to assess the detection performance. FoM is defined as [28]

(26) $$FoM=\frac{N_{tt}}{N_{fa}+N_{gt}}$$

where $N_{tt}$ is the number of correctly detected targets, $N_{fa}$ is the number of false alarms, and $N_{gt}$ is the number of true targets that existed in image.

The higher value of FoM means higher detection rate and lower false alarm rate. The comparison of FoM before and after sea spike suppression on sub-block C is shown in Table 4.

Comparing the values of FoM before and after sea spike suppression, it can be found that after sea spike suppression, the false alarm rate of ship detection is greatly reduced, which fully demonstrates the effectiveness of this method.

## 4. Discussion

### 4.1. Comparison Between the Proposed Method and Spectrum Filtering Method

**Figure 15:**
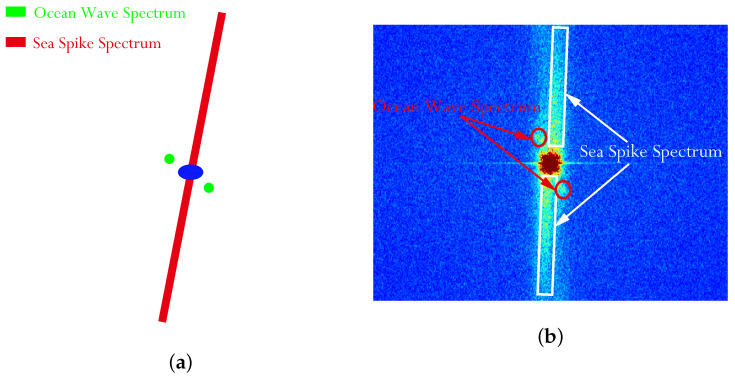
Distribution of sea surface components in the spectral domain of SAR image. (**a**) Schematic diagram of sea surface spectrum. (**b**) SAR image spectrum of sea surface.

At present, there is no report on the sea spike suppression method in SAR images. In this paper, referring to the spectrum analysis method of ship wake components [29], we try to use the spectrum filtering method to suppress the sea spikes in SAR images. Through the field data analysis, it is found that the spectrum distribution of the sea spikes is shown in the red area of Figure 15a, and the corresponding SAR image spectrum is shown in the white area of Figure 15b. The spectrum filtering is used to remove the sea spike spectrum of the VV polarized and HH polarized SAR images. The three aspects, namely ocean wave contrast, oil spill contrast and ship detection results, are compared with those of the proposed method.

#### 4.1.1. Comparison of the Ocean Wave Contrast

**Figure 16:**
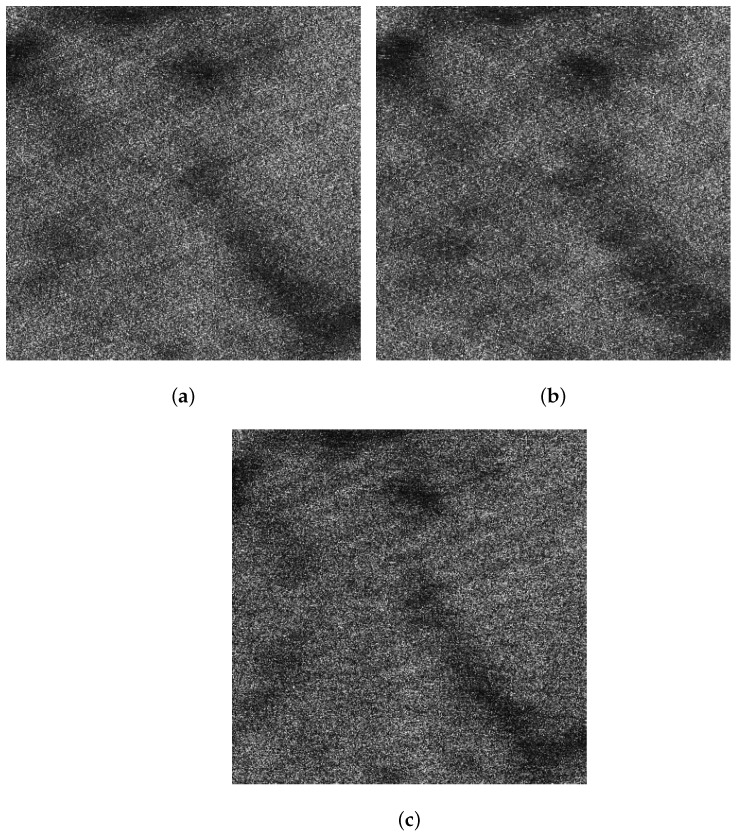
Comparison of results of sub-block A. (**a**) The VV polarized SAR image after spectrum filtering. (**b**) The HH polarized SAR image after spectrum filtering. (**c**) The image after the proposed method processing.

In this subsection, the results of the proposed method and the spectrum filtering method corresponding to sub-block A are compared. The VV polarized SAR image and the HH polarized SAR image are shown in Figure 16a,b after spectrum filtering. The image after the proposed method processing in this paper is shown in Figure 16c.

Compared with the spectrum filtering method, the texture of the ocean waves obtained by the proposed method in this paper is clearer. The following is a quantitative analysis by calculating PBR. The comparison of PBR of spectrum filtering method and the proposed method is shown in Table 5.

Compared with the spectrum filtering method, the PBR is the largest after the sea spike suppression by the proposed method in this paper, indicating that the contrast of the ocean waves is highest. At the same time, comparing Table 2 and Table 5, it can be found that the PBR of VV polarized SAR image corresponding to sub-block A is slightly improved than the original image, but the PBR of HH polarized SAR image is significantly reduced. The reason is that the ocean waves information of the HH polarized SAR image is lost after the spectrum filtering.

#### 4.1.2. Comparison of the Oil Spill Contrast

**Figure 17:**
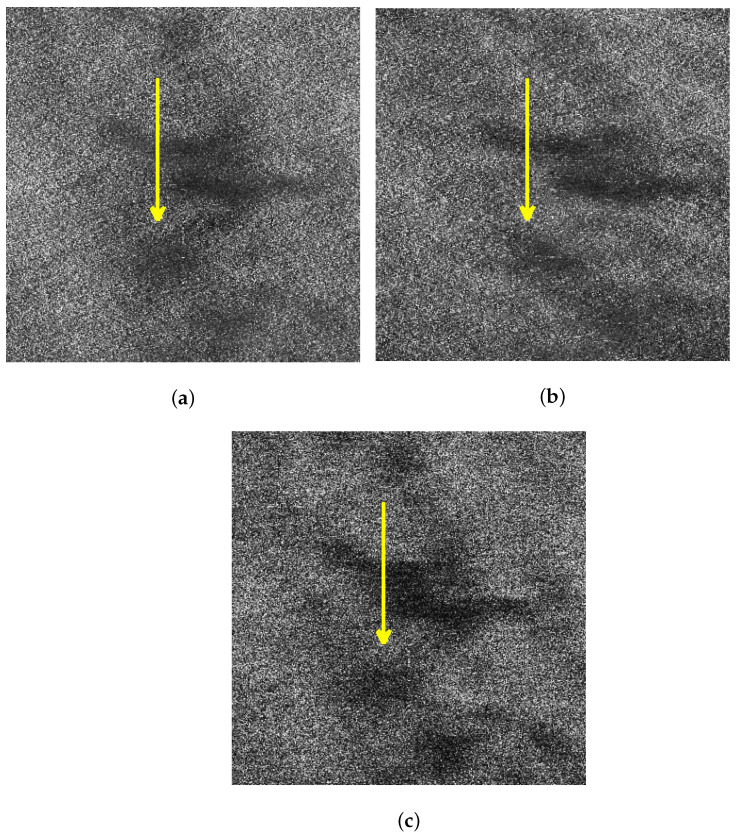
Schematic diagrams of the oil spill profiles in sub-block B. (**a**) Schematic diagram of the VV polarized oil spill profile after spectrum filtering. (**b**) Schematic diagram of the HH polarized oil spill profile after spectrum filtering. (**c**) Schematic diagram of the oil spill profile after the proposed method processing.

**Figure 18:**
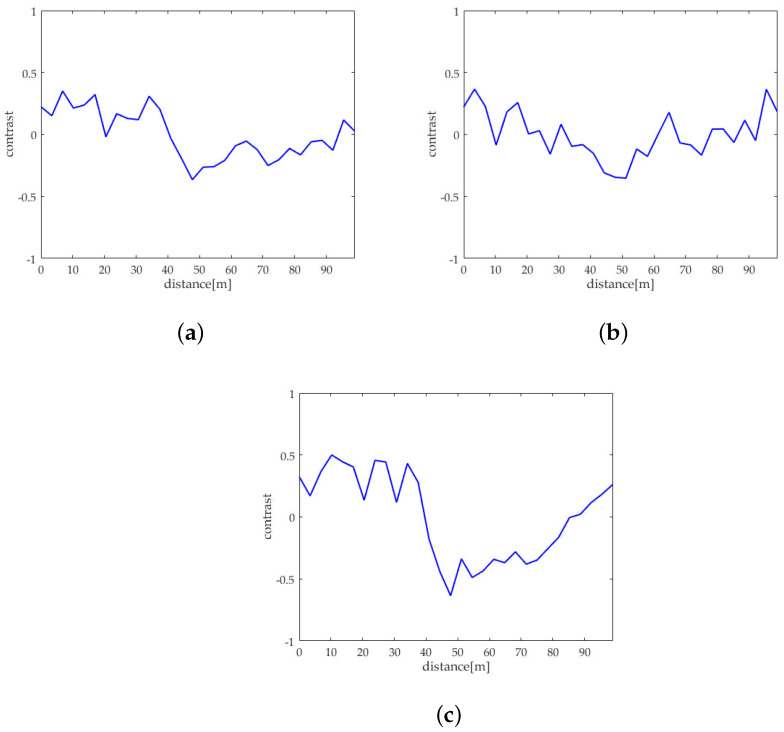
Comparison of contrasts of the oil spill profiles. (**a**) The contrast of the VV polarized oil spill profile after spectrum filtering. (**b**) The contrast of the HH polarized oil spill profile after spectrum filtering. (**c**) The contrast of the oil spill profile after the proposed method processing.

In this subsection, the results of the proposed method and the spectrum filtering method corresponding to sub-block B are compared. The VV polarized SAR image and the HH polarized SAR image are shown in Figure 17a,b after spectrum filtering. The image after the proposed method processing of this paper is shown in Figure 17c. Calculating the contrast of profiles shown along the yellow arrows in Figure 17a–c, the obtained contrasts corresponding to the profiles are shown in Figure 18a–c, respectively.

Comparing Figure 18a–c, it can be found that the contrast of the oil spill profile after the proposed method processing is higher than spectrum filtering. Calculating the value of ξ in the profiles shown in Figure 17, the comparison of ξ of spectrum filtering method and the proposed method is shown in Table 6.

As can be seen from Table 6, the oil spill contrast after the proposed method processing is higher than that of the spectrum filtering results. It demonstrates the superiority of the proposed method in this paper compared with the spectrum filtering method.

#### 4.1.3. Comparison of Ship Detection Results

**Figure 19:**
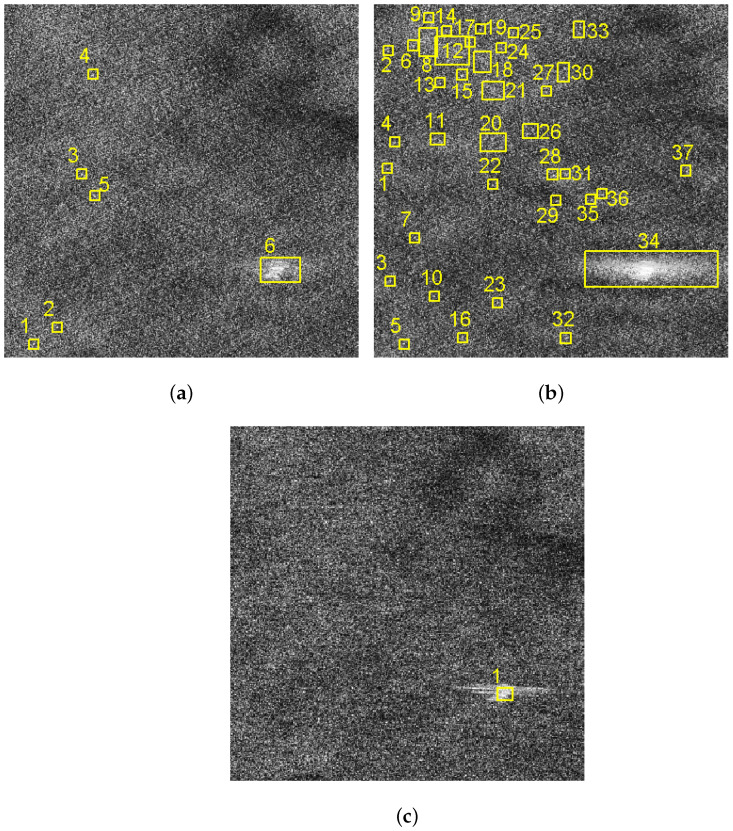
Comparison of ship detection results of images of sub-block C. (**a**) Ship detection result of the VV polarized SAR image after spectrum filtering. (**b**) Ship detection result of the HH polarized SAR image after spectrum filtering. (**c**) Ship detection result after the proposed method processing.

In this subsection, the ship detection results of the proposed method and the spectrum filtering method corresponding to sub-block C are compared. The ship detection results of VV polarized SAR image and the HH polarized SAR image are shown in Figure 19a,b after spectrum filtering. The ship detection result after the proposed method processing of this paper is shown in Figure 19c.

By the comparison, it can be found that the false alarm rate of the ship detection is higher after spectrum filtering method. The comparison of FOM of spectrum filtering method and the proposed method is shown in Table 7.

As can be seen from Table 7, the false alarm rate after the proposed method processing is lower than that of the spectrum filtering results, which fully demonstrate the superiority of the proposed method in this paper. At the same time, comparing the results of Table 4 and Table 7, it can be found that the spectrum filtering method will lead to rising in the false alarm rate of ship detection. The main reason is that some strong interference points appear after the spectrum filtering.

### 4.2. Analysis of the Applicability of the Proposed Method

This paper fully demonstrates that the method in this paper can effectively suppress sea spikes through three aspects: analysis of ocean wave contrast, analysis of oil spill contrast and analysis of ship detection result, but this method still has some shortcomings.

**Figure 20:**
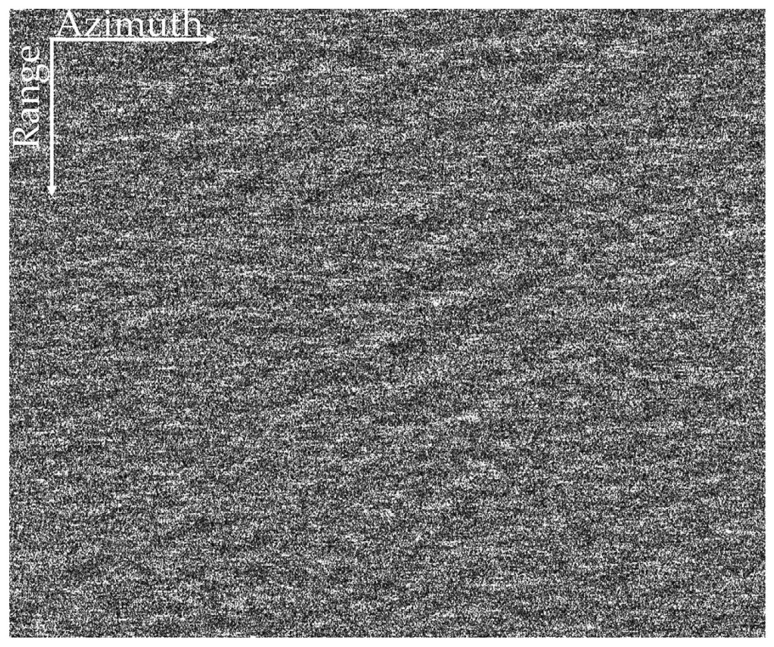
Image of TerraSAR-X HH polarization.

(1) The method in this paper is based on data of SAR observing the ocean. On the one hand, the method in this paper is only applicable to the situation where sea spikes appear during SAR observing the ocean. For low frequency SAR, especially P, L band, SAR images usually do not contain sea spikes, so sea spike suppression is not required. On the other hand, the VV and HH polarized SAR data must be obtained at the same time.

(2) The method in this paper requires that a uniform background sub-block containing only sea spikes can be selected from the VV and HH polarized SAR images. For SAR images almost completely covered by man-made targets such as ships and oil spills, a uniform background sub-block only containing sea spikes cannot be selected from them, the optimum polarization ratio cannot be calculated. The method is not applicable.

(3) Theoretically, the proposed method can be well applied to the spaceborne dual-polarized SAR data that are high-resolution and contain sea spikes. For example, as shown in Figure 20, sea spikes is obvious in the HH polarized image obtained by TerraSAR-X around Australia in 2010. However, since there is no VV polarized data obtained at the same time, the method in this paper has not been applied to spaceborne SAR field data for processing verification.

## 5. Conclusions

Sea spikes are prone to appear when high-resolution SAR is used to observe the ocean, causing serious interference to SAR image interpretation and marine target detection. Aiming at the problem that there is no report on sea spike suppression methods in SAR images, a sea spike suppression method based on optimum polarization ratio in airborne SAR images is proposed in this paper. This method generates a sequence of polarization ratios by traversing the theoretical interval of the polarization ratio. The sequence of difference images of background sub-block BK corresponding to the sequence of polarization ratios is calculated. The sequence of difference images of the background sub-block is compared to obtain the optimum polarization ratio, and finally the sea spike suppression is achieved by calculating the panoramic difference image. The method in this paper is applied to X-band airborne SAR data, and the image of sea spike suppression is obtained. It is discussed and analyzed through three aspects: ocean wave contrast, oil spill contrast, and ship detection results. After processing by this method, the results show that the PBR of ocean wave contrast, the ξ of oil spill contrast, and the FoM of ship detection results are greatly improved compared with dual polarized SAR images. Through the analysis of these three aspects, the effectiveness of the method in this paper is fully demonstrated, and the foundation is laid for the subsequent target detection and parameter inversion.

Moreover, the proposed method and the spectrum filtering method are compared. On the one hand, due to the aliasing of the spectrum of other ocean phenomena and sea spikes, the spectrum filtering method will cause the loss of other ocean phenomenon information. On the other hand, the spectrum filtering method is not fully utilized polarization information, so the effect is not as obvious as the results of the proposed method in this paper, which fully illustrates the superiority of the proposed method.

Finally, the applicability of the method in this paper is discussed. (1) The method in this paper is only applicable to the situation where sea spikes appear during SAR observing the ocean. Besides, the VV and HH polarized SAR data must be obtained at the same time. (2) A uniform background sub-block containing only sea spikes can be selected from the VV and HH polarized SAR images. (3) It is stated that this method is still applicable for high-resolution spaceborne dual-polarized SAR data containing sea spikes, but it has not been applied to spaceborne SAR field data for processing verification due to the limitation of the data. In the follow-up, a large amount of data processing is necessary for more in-depth analysis, such as the discussion of the relationship between the optimum polarization ratio and sea state.

## Figures and Tables

**Table 1:** Parameters of the SAR system.

Parametric Name	Parametric Symbol	Parametric Value
Radar wavelength (m)	λ	0.0313
Pulse length (us)	$T_{r}$	6
Radar bandwidth (MHz)	$B_{r}$	606
Platform speed (m/s)	*V*	74.65
PRF (Hz)	PRF	2000
Platform height (m)	*H*	3409

**Table 2:** Comparison of PBR before and after sea spike suppression on sub-block A.

	VV Polarization	HH Polarization	Sea Spike Suppression Result
PBR	5.2426	3.0235	7.5365

**Table 3:** Comparison of ξ before and after sea spike suppression on sub-block B.

	VV Polarization	HH Polarization	Sea Spike Suppression Result
ξ	1.7688	1.3909	1.8756

**Table 4:** Comparison of FoM before and after sea spike suppression on sub-block C.

	VV Polarization	HH Polarization	Sea Spike Suppression Result
FoM	0.33	0.08	1

**Table 5:** Comparison of PBR of spectrum filtering method and the proposed method.

	VV Polarization Result after Spectrum Filtering	HH Polarization Result after Spectrum Filtering	Reslut after the Proposed Method Processing
PBR	5.6547	1.3039	7.5365

**Table 6:** Comparison of ξ of spectrum filtering method and the proposed method.

	VV Polarization Result after Spectrum Filtering	HH Polarization Result after Spectrum Filtering	Reslut after the Proposed Method Processing
ξ	1.2775	1.3641	1.8756

**Table 7:** Comparison of FOM of spectrum filtering method and the proposed method.

	VV Polarization Result after Spectrum Filtering	HH Polarization Result after Spectrum Filtering	Reslut after the Proposed Method Processing
FOM	0.17	0.03	1

## Data Availability

Data sharing not applicable.
